# Comparison of the repair of potentially lethal damage after low- and high-LET radiation exposure, assessed from the kinetics and fidelity of chromosome rejoining in normal human fibroblasts

**DOI:** 10.1093/jrr/rrt031

**Published:** 2013-05-14

**Authors:** Cuihua Liu, Tetsuya Kawata, Guangming Zhou, Yoshiya Furusawa, Ryuichi Kota, Atsuhiro Kumabe, Shinya Sutani, Junichi Fukada, Masayo Mishima, Naoyuki Shigematsu, Kerry George, Francis Cucinotta

**Affiliations:** 1 Research Center for Charged Particle Therapy, National Institute of Radiological Sciences, Chiba 263-8555, Japan; 2 Department of Radiology, School of Medicine, Keio University, Tokyo, 160-8582, Japan; 3 Department of Space Radiobiology, Institute of Modern Physics, Chinese Academy of Sciences, Lanzhou 730-000, China; 4 Wyle Integrated Science and Engineering Group, Houston, Texas, USA; 5 NASA Johnson Space Center, Radiation Biophysics, Houston, Texas, USA

**Keywords:** heavy ion, PLDR (potentially lethal damage repair), premature chromosome condensation, FISH (fluorescence *in situ* hybridization), misrepair

## Abstract

Potentially lethal damage (PLD) and its repair (PLDR) were studied in confluent human fibroblasts by analyzing the kinetics of chromosome break rejoining after X-ray or heavy-ion exposures. Cells were either held in the non-cycling G_0_ phase of the cell cycle for 12 h, or forced to proliferate immediately after irradiation. Fusion premature chromosome condensation (PCC) was combined with fluorescence *in situ* hybridization (FISH) to study chromosomal aberrations in interphase. The culture condition had no impact on the rejoining kinetics of PCC breaks during the 12 h after X-ray or heavy-ion irradiation. However, 12 h after X-ray and silicon irradiation, cycling cells had more chromosome exchanges than non-cycling cells. After 6 Gy X-rays, the yield of exchanges in cycling cells was 2.8 times higher than that in non-cycling cells, and after 2 Gy of 55 keV/μm silicon ions the yield of exchanges in cycling cells was twice that of non-cycling cells. In contrast, after exposure to 2 Gy 200-keV/μm or 440-keV/μm iron ions the yield of exchanges was similar in non-cycling and cycling cells. Since the majority of repair in G_0_/G_1_ occurs via the non-homologous end joining process (NHEJ), increased PLDR in X-ray and silicon-ion irradiated cells may result from improved cell cycle-specific rejoining fidelity through the NHEJ pathway, which is not the case in high-LET iron-ion irradiated cells.

## INTRODUCTION

If cells are held in the non-cycling phase (G_0_) for several hours (delayed plating, DP) after X-ray or γ-ray irradiation their survival will be greater than if they are forced to proliferate immediately (immediate plating, IP) after exposure [1–3]. Some reports suggest that proliferative conditions preserve the potentially lethal damage (PLD) [1–7]. Therefore, comparisons of non-cycling and proliferating cells can provide a measure of PLD and potentially lethal damage repair (PLDR). When the same initial yield of double-strand breaks (DSB) is induced, any difference in survival rate between cycling and non-cycling cells could be due to differences in the number of DSBs that are either misrejoined or remain unrejoined. Since chromosomal aberrations result from misrepair of DSBs, whole chromosome FISH (fluorescence *in situ* hybridization) analysis provides useful information concerning misrejoined and unrejoined breaks under PLD and PLDR conditions.

Previously we studied normal human fibroblasts, that were subcultured immediately or 24 h after irradiation, and chromosome damage was assessed in the first post-irradiation G_2_ phase of the cell cycle using a Calyculin-A-induced PCC (premature chromosome condensation) technique [8]. Results reveal lower yields of inaccurate chromosome repair when normal fibroblast cells are held under non-cycling conditions than when they are forced into the cell cycle immediately after X-ray irradiation. However, Tenhumberg *et al.* reported that permanent G_1_ arrest is prevalent in primary human fibroblasts and increases with radiation dose [9]. It has also been reported that the fraction of permanent G_1_ arrest is substantially higher in cells that are forced to cycle immediately after irradiation than in cells held in G_0_ for extended times [10–12]. Thus, limiting assessment of damage to the G_2_-phase of the cell cycle would underestimate the true yield of total chromosome damage in first division after irradiation exposure.

Frankenberg-Schwager *et al.* studied the mechanisms of PLDR, using a plasmid-mediated assay in yeast cells and demonstrated the enhanced fidelity of DSB rejoining under non-growth conditions compared to active growth conditions [13]. In a previous study using G_0_/G_1_ PCC and FISH analysis we demonstrated that in normal fibroblast cells enhanced repair fidelity under non-cycling conditions accounted for increased PLDR after X-ray irradiation [1]. Several studies have been conducted to assess the effects of high-LET radiation on PLDR. Blakely *et al.* reported that delayed plating after X-irradiation resulted in significant PLDR and survival increased up to 10-fold in a dose-dependent manner, whereas there was negligible PLDR in early and mid G_1_-phase cells after neon ion exposures and only late G_1_-phase cells repaired neon damage [14]. In addition Suzuki *et al.* reported that the recovery ratio of the PLDR was dependent on the quality of radiation [15]. Autsavapromporn *et al.* reported that low-LET radiation induced strong PLDR within hours, whereas high-LET radiation at similar immediate toxicity levels did not induce PLDR, and toxicity increased with post-irradiation time [16].

In the present study we extended our previous work on X-rays and have included analysis of high-LET radiation using a fusion PCC and FISH method to study the chromosome break rejoining kinetics and fidelity of DSBs induced in the G_0_/G_1_ phase of the cell cycle. Non-cycling (G_0_) human fibroblasts (AG01522) were exposed to 6 Gy of X-rays or 2 Gy of Si or Fe heavy ions, and subsequently the cells were either allowed to repair in G_0_ phase or were immediately stimulated to begin cycling. After incubation, PCC samples were collected from both cultures at different times using the viral fusion method. This method forces chromosomes to condense in interphase, allowing the frequency of unrejoined PCC breaks to be compared in non-cycling cells at G_0_ phase and those cycling at G_1_. We then assessed chromosome damage using FISH, a technique that facilitates accurate analysis of misrejoined chromosomes [1, 17–21]. Repair efficiency and fidelity were thereby directly assessed in non-cycling G_0_ and cycling G_1_ cells while avoiding the complications induced by permanent G_1_ cell cycle arrest.

## MATERIALS AND METHODS

### Cells and cell culture

AG01522 normal human diploid skin fibroblasts were obtained from the NIA Aging Cell Repository. Low-passage AG01522 cells were maintained in minimum essential medium (MEM) supplemented with 15% fetal bovine serum and antibiotics at 37°C in an atmosphere of 5% CO_2_ and 95% air. Cells (passage 12–14) were plated into T25 flasks at 25% confluence and grown for 7 days before being irradiated in the confluent state. The cells were counted at the time of irradiation and 24 h after exposure with a Coulter counter. No significant changes in cell number were observed, indicating that most of the cells were not cycling while in the confluent state.

### Irradiations

The confluent AG01522 fibroblasts in T25 plastic flasks (Nunc 152094) were irradiated at room temperature at a dose rate of 2 Gy/min using an MBR-1520R X-rays device (Hitachi Medical: 150 kV and 20 mA with a 1-mm aluminum shielding) or irradiated with silicon or iron ions accelerated by the Heavy Ion Medical Accelerator in Chiba (HIMAC) at the National Institute of Radiological Sciences (NIRS), Japan. The initial energy of the silicon-ions was 490 MeV/u with an average LET value of 55 keV/µm, and the energies of the iron ions were 200 MeV/u and 500 MeV/u, with corresponding LET values of 440 keV/µm and 200 keV/µm, respectively.

The details of the HIMAC beam-delivery system, physical characters, biological irradiation procedures, and dosimetry have been described elsewhere [22–23]. Before irradiation, the cells were kept on ice to prevent repair during the exposure period. Cells were either allowed to repair at G_0_ phase or subcultured immediately after radiation and samples collected at different times until 12 h.

### Clonogenic survival

Cell survival was assessed by the frequency of colony formation. Immediately or 12 h after irradiation, cells were trypsinized and different numbers of cells dependent on radiation dose, were plated into 100-mm-diameter plastic dishes. The cells were incubated for 14 days at 37°C and then fixed with 100% methanol and stained with 0.2% crystal violet. Survival rates were derived from the number of colonies containing ≥ 50 cells in comparison with the initial number of plated cells.

### Induction of PCC

The PCC method is based on previous methods with a few modifications [1, 18–19, 24–25]. Cells were fused with mitotic HeLa cells to prematurely condense chromosomes in G_0_ or G_1_. Briefly, 1 × 10^6^ irradiated cells were mixed with 1 × 10^6^ HeLa mitotic cells (mitotic index >95%, frozen and thawed) in ice-cold media. The cells were centrifuged at 1500 rpm for 5 min and cell pellets were washed in ice-cold serum-free media, then immediately treated with 2–4 μl of hemagglutinating virus of Japan envelope (HVJ-E; also known as Sendai virus) (Ishihara Sangyo, Japan). The HVJ-E-treated cells were kept on ice for 15 min to allow the virus envelope to attach and were then placed in a water bath at 37°C for 3 min. The samples were then incubated at 37°C to allow cell fusion and PCC induction to occur. After 1 h of incubation, the samples were carefully resuspended in 8 ml of 75 mM KCl. After a 20 min incubation at 37°C, 2 ml of freshly prepared fixative solution (methanol: glacial acetic acid = 3:1 vol/vol) was slowly added to the solution, and the cells were centrifuged again. After two further washes in fixative solution, chromosomes were dropped onto wet slides at room temperature and air-dried. In cases where studies were limited to the measurement of excess fragments only (i.e. gross breakage), the cells were then stained with 5% Giemsa and PCC were analyzed under a light microscope.

### FISH analysis

Slides containing PCC were aged overnight at 37°C and hybridized *in situ* with fluorescent whole chromosome painting probes 1 (Spectrum green) and 3 (Spectrum red) (Vysis USA) using the procedures recommended by the manufacturer. Cells were counterstained with DAPI and viewed with a Zeiss Axioskop fluorescence microscope. The fraction of aberrant cells was calculated as the ratio of the number of cells containing one or more aberrations involving chromosomes 1 and/or 3 to the total number of cells analyzed.

### Scoring of chromosome aberrations

#### Giemsa-stained PCC samples

The frequencies of Giemsa-stained PCC breaks were determined from the number of excess chromosome fragments. First the total number of chromosome fragments was counted and then 46 was subtracted from the value to give the number of excess chromosome fragments. At least 50 cells were assessed for each datapoint. The data represent all types of excess fragments, and no attempt was made to distinguish between linear or circular fragments. The excess fragments in unirradiated AG01522 cells were negligible (data not shown).

#### FISH-painted PCC samples

Damage was assessed in chromosomes 1 and 3. No damaged chromosomes were detected in the non-irradiated controls. After irradiation, one or both of the chromosomes 1 or 3 might be broken and any number of the broken ends could be involved in an exchange (color-junction) with non-painted chromosomes or other FISH-painted chromosomes. Therefore an exchange event was defined as any bicolor-junction between chromosomes 1 or 3 and/or between the painted chromosomes and the DAPI-stained chromosomes; each bicolor-junction was counted as one event [26]. The number of color-junctions per cell is a simple parameter representing the frequency of chromosome misrejoining, and the number of excess painted fragments represents non-rejoined breaks. These excess fragments, which would presumably include both interstitial and terminal-type deletion, were included in a single category of deletions. The percentage of aberrant cells, which gives a direct measurement of the extent of chromosome damage, was calculated as the radio of the number of aberrant cells and the total number of cells scored. A minimum of 45 aberrant cells were analyzed for each data point from one experiment. The total number of cells analyzed for each data point ranged from 45 to 90.

### Flow cytometry analysis

Flow cytometry analysis of DNA content was used to assess the cell cycle distribution with incubation time. Briefly, irradiated cells were rinsed twice with PBS, trypsinized, and plated at a density of about 1.5 × 10^6^ onto 100-mm dishes and incubated at 37°C in an atmosphere of 5% CO_2_ and 95% air for a desired time. At different incubation times after subculture, the cells were collected by trypsinization and fixed with 70% ethanol. The cellular RNA was digested with 0.5 mg/ml ribonuclease (90 U/mg, Roth) for 30 min at 37°C. The cells were centrifuged, rinsed twice in PBS, and resuspended in 1 ml of PBS with propidium iodide (0.5 mg/ml). The cells were filtered through a 30-µm nylon mesh to remove debris and analyzed on a flow cytometer (Becton-Dickinson, FACS Carlibar). The DNA histograms were analyzed graphically to determine the populations of G_0_/G_1_, S, and G_2_/M cells.

## RESULTS

### Comparison PLDR from cell surviving fraction irradiated by X-ray and different heavy ion beams

**Fig. 1.**
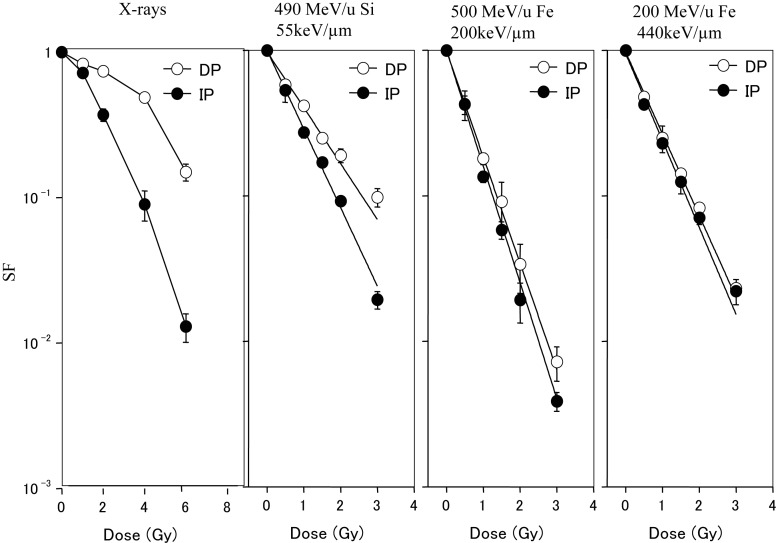
Survival fractions of confluent normal AG01522 fibroblasts irradiated with X-rays or heavy ions [1]. The closed circle shows the survival of cells subcultured immediately after irradiation (IP). The open circle shows that of cells allowed to repair for 12 h and then subcultured (DP).

Figure 1 shows the dose–survival curves for AG01522 cells that were plated either immediately or 12 h after X-ray or heavy-ion irradiation. Markedly increased survival was observed in the cells that were allowed to repair for 12 h after X-ray exposure. When cells were irradiated with silicon ions (490 MeV/u, LET: 55 keV/µm), the survival fraction after 12 h incubation was also higher than cells plated immediately after radiation. In contrast, after 200 MeV/u and 500 MeV/u iron ion exposures (LET: 200 keV/µm, 440 keV/µm, respectively) delayed plating resulted in almost no change in survival.

### Cell cycle distribution

**Fig. 2.**
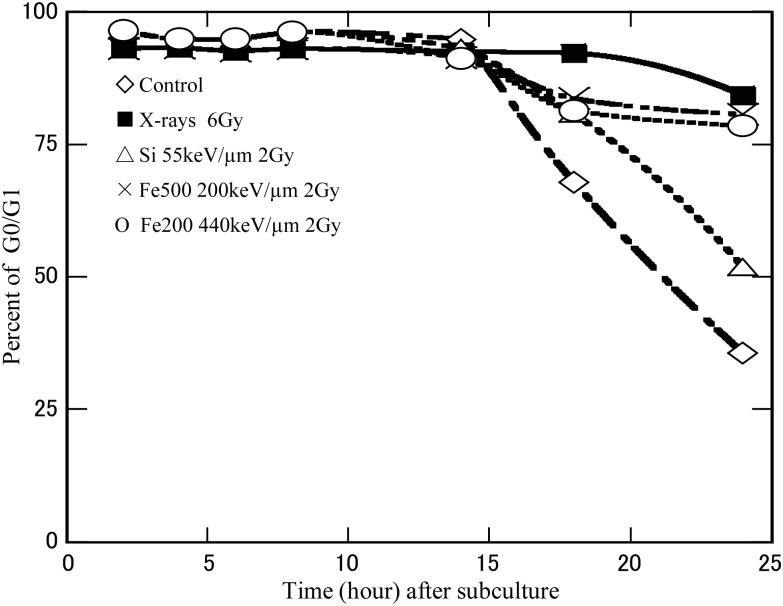
The percentage of the cell population in G_0_/G_1_ population at different incubation times for cells subcultured immediately after 0 Gy (open diamonds), 6 Gy X-rays (closed squares), and 2 Gy 55 keV/µm silicon-ion radiation (open triangles), and 2 Gy 200 keV/µm (Xs) and 440 keV/µm (open circles) iron-ion radiation. The dashed line indicates 12 h after subculture.

Figure 2 shows the cell cycle distributions of non-irradiated cells and irradiated cells at different incubation times after subculture. The data, which was obtained by flow cytometry, revealed more than 93% of the cell population was in G_0_/G_1_ phase at the time of exposure. For all exposures types, the percentage of G_0_/G_1_ phase cells was essentially constant for the first 14 h after being subcultured. The cells began to enter S or G2/M phase about 18 h after subculture with a greater percentage of S or G2/M phase cells in the non-irradiated compared to the irradiated cells.

### Repair kinetics of chromosome breaks after low- or high-LET irradiation in non-cycling and cycling cells

**Fig. 3.**
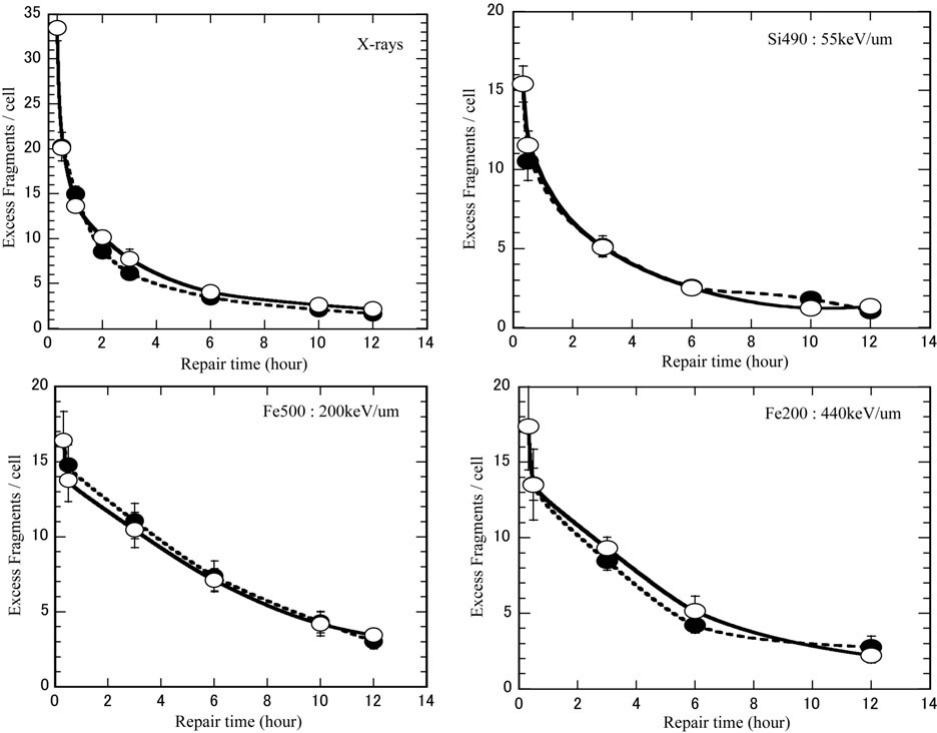
Kinetics of repair of prematurely condensed chromosome breaks under non-cycling and cycling conditions irradiated by X-ray, Si 490 MeV/u (55 keV/µm), Fe 500 MeV/u (200 keVµm), Fe 200 MeV/u (440 keV/µm) ion beams. The data were obtained after Giemsa staining. Closed symbols represent non-cycling cells, open symbols represent cycling cells. Bars are the standard errors of the mean values.

We studied the time-course of chromosome break rejoining and identified the number of unrejoined chromosome fragments within 12 h of irradiation. Figure 3 shows the repair kinetics of chromosome breaks under non-cycling (PLDR) and cycling (PLD) conditions after irradiation with X-rays, silicon, or iron ions. Since some repair inevitably occurs during the period of cell fusion and chromosome condensation, the earliest sampling time immediately after irradiation was estimated at 0.33 h, a time period that has been suggested as a best estimate of the time taken for cells to fuse and for DNA to sufficiently condense to prevent further break rejoining [25]. Under both culture conditions, the number of G_0_/G_1_ Giemsa-stained PCC fragments was measured after different repair times. The results indicated that the efficiency of rejoining PCC fragments was similar for non-cycling G_0_ and cycling G_1_ phase, regardless of radiation type. The X-ray data in Fig. 3 has been published previously [1].

### Chromosome aberration assay

**Fig. 4.**
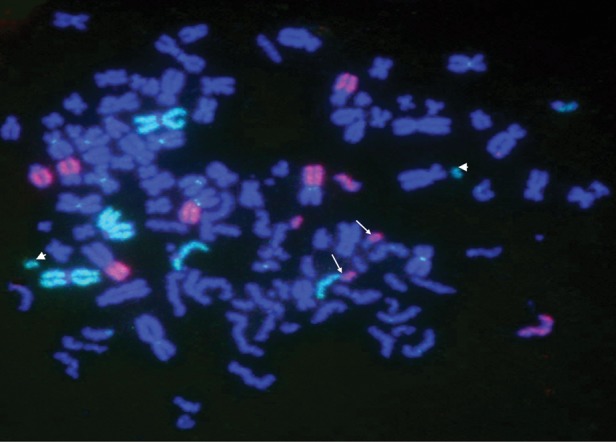
An example of a cell with chromosomal exchanges FISH sample. The green indicates chromosome 1, the red chromosome 3. The arrows show the color-junction, the arrowheads show the fragment no repaired.

The FISH technique with whole chromosome painting probes was used for detecting the fidelity of rejoining in non-cycling and cycling cells. An example of a cell with exchanges is shown in Fig. 4, two one bi-color-junction events in chromosome 1 (arrow) and two fragments in chromosome 3 (arrowhead) were identified in this cell.

**Fig. 5.**
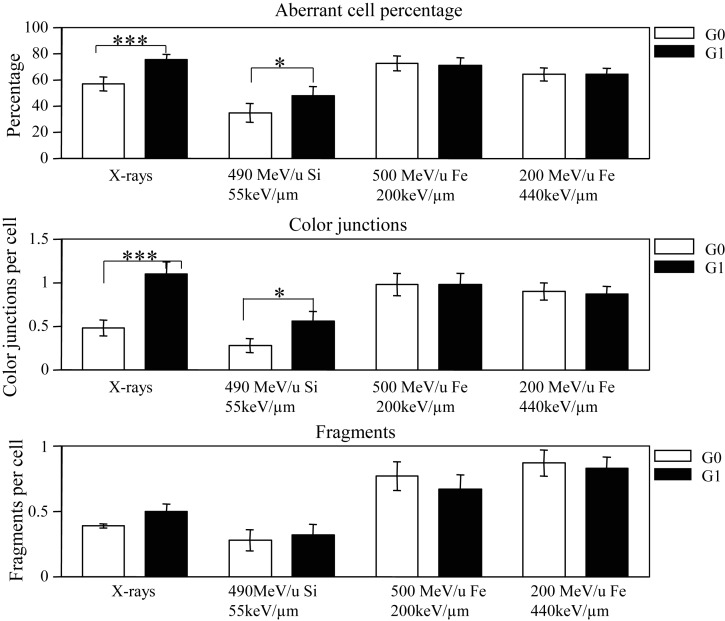
Chromosome aberrations in human fibroblast AG01522 cells exposed to X-rays (6 Gy), Si490 (2 Gy), Fe500 (2 Gy) and Fe200 (2 Gy) analyzed by FISH painting. Aberrant cell percentage, color-junction per cell and fragments per cell in non-cycling G_0_ and cycling G_1_ cells after 12-h incubation were shown. Open bars show G_0_ chromosome aberrations (delayed plating) and closed bars show G_1_ aberrations (immediate plating). The error bars are standard errors of the means. A statistically significant difference between non-cycling G0 and cycling G1 phase cells is indicated by *(*P* < 0.05), ***(*P* < 0.005).

The fidelity of rejoining under non-cycling and cycling conditions were measured 12 h after irradiation and the results are shown in Fig. 5. The upper panel in Fig. 5 shows the percentages of aberrant cells with fragments and/or exchanges after 12 h incubation. Aberrations were detected in 76% of cycling cells and 57% of non-cycling cells after a 6-Gy exposure of X-rays (*P* < 0.005), compared with 48% of cycling cells and 35% of non-cycling cells (*P* < 0.05) after a 2-Gy exposure of 490 MeV/u silicon ions (LET: 55 keV/µm). The percentages of aberrant cells in cycling conditions were about 1.3 times and 1.4 times higher than that under non-cycling conditions for X-rays and silicon ions, respectively. However, after exposure to 500-MeV/u and 200-MeV/u iron ions (LET: 200 keV/µm and 440 keV/µm), cycling and non-cycling cells have almost the same yield of damaged cells.

The middle panel in Fig. 5 shows the yield of color-junctions per cell for non-cycling and cycling cells. After 6 Gy X-rays, an average of 1.26 color-junctions per cell was detected in cycling cells compared with just 0.48 in non-cycling cells (*P* < 0.005), 2.8 times higher than that in non-cycling cells. After 2 Gy of silicon ions the yield of color-junctions in cycling cells double the yield in non-cycling cells (*P* < 0.05). In contrast, 2 Gy of iron ions induced almost the same yields of color-junctions in non-cycling and cycling cells.

The bottom panel in Fig. 5 shows the average yields of fragments per cell in non-cycling and cycling cells. Culture condition had almost no effect on the yield of fragments for any of the radiation types.

## DISCUSSION

The aim of the present study is to clarify the relationship between PLDR and the repair efficiency and fidelity of chromosome break rejoining in normal human fibroblasts after X-ray and heavy-ion exposure. When confluent normal fibroblast AG01522 cells were irradiated with X-rays or heavy-ion beams and plated either immediately or 12 h after exposure, significantly increased cell survival was observed in the cells that were allowed to repair for 12 h after X-ray or silicon (LET: 55keV/µm) heavy ion exposure. In contrast, almost no change in survival was observed for the two culture conditions after 200 MeV/u or 500 MeV/u iron ion exposures (LET: 440 keV/µm, 200 keV/µm), respectively (Fig. 1). These results may imply that PLDR occurs after X-rays and ions in the mid- to lower-LET range, whereas almost no PLDR occurs after high-LET radiation exposure. Our results are agreement with the report by Blakely *et al.* that the repair capacities appear to be LET-dependent [14].

About 93% of normal fibroblast cells were in G_0_/G_1_ phase during irradiation in confluent conditions. During the first 14 h after sub-culturing the percentage of G_0_/G_1_ cells was similar for all irradiated and non-irradiated samples. It was not until 18 h after subculture that the populations diverged and more G_0_/G_1_ cells were observed in the irradiated samples than in the non-irradiated samples, probably due in part to G_1_ cell cycle arrest or permanent G1 arrest in the irradiated cells. A direct assessment of repair requires comparison of damage in the G_1_ phase of cycling cells with damage in the G_0_ phase of non-cycling cells. Since the fusion-PCC technique can provide a direct assessment of chromosome repair efficiency during non-cycling G_0_ and cycling G_1_ phases, we applied this method to study the repair efficiency under non-cycling and cycling conditions (Fig. 3). DNA double-strand breaks are the major lethal event in irradiated cells and it is possible that the lack of PLDR in iron ion-irradiated samples may be due to non-repairable DNA damage in these cells [27]. However, our results indicated that the efficiency of rejoining PCC fragments was similar for non-cycling G_0_ and cycling G_1_ phase, regardless of radiation type (Fig. 3), and this implies that the lack of PLDR in iron ion-irradiated cells is not due to non-repairable DNA damage. This data supports work by Wolff *et al.*, who reported no difference in the yield of radiation-induced chromosome breaks between non-stimulated (G_0_) and stimulated (G_1_) human lymphocytes after repair [17]. Moreover, similar break rejoining kinetics in G_0_ and G_1_ (Fig. 3) also fails to explain why cell survival is enhanced by holding cells in G_0_ after X-ray and 55 keV/µm silicon ion exposure. To the best of our knowledge, the kinetics of chromosomal repair in the cycling human fibroblasts sub-cultured immediately after heavy-ion beam irradiation has never been reported.

We subsequently assessed the fidelity of chromosomal break rejoining using PCC combined with the FISH technique in non-cycling G_0_ and cycling G_1_ phase cells after 12 h incubation. The results showed 76% of cycling cells and 57% of non-cycling cells contained aberrations (unrepaired chromosome fragments and/or chromosome exchanges) after a 6-Gy exposure of X-rays, and 48% of cycling cells and 35% of non-cycling cells contained aberrations after a 2-Gy exposure of 55 keV/µm silicon ions. This contrasts with the results of exposure to higher-LET iron ions (200 keV/µm and 440 keV/µm), where cycling and non-cycling cells had almost the same yield of damaged cells (Fig. 5). After 6 Gy of X-rays the yield of chromosome exchanges was 2.8 times higher in cycling G_1_ phase than in non-cycling G_0_ phase, and 2 times higher after 2 Gy of 55 keV/µm silicon-ion radiation. However, almost the same yield of color-junctions was observed in cycling G_1_ and non-cycling G_0_ phase after 2 Gy of iron-ion beams (200 keV/µm and 440 keV/µm). Therefore, it is possible that an enhanced survival after extended incubation at G_0_ after exposure to X-rays or 55 keV/µm silicon ions is due to an enhanced fidelity of DSBs or chromosomal break rejoining under the non-cycling G_0_ condition relative to the cycling G_1_ condition. Whereas, after exposure to the higher-LET iron ions, impaired fidelity of chromosome breaks is similar in non-cycling and cycling cells, resulting in survival fractions that are almost the same.

Krüger *et al.* indicated that confluent non-cycling G_0_ cells may have exited the cell cycle and possibly could possess a state of chromosome condensation different from cycling G_1_ phase cells [28].

Pantelias also reported that the repair mechanism was dependent on cellular metabolism, energy production, and protein synthesis. Apparently, chromosome decondensation interferes with repair processes [29]. The cells we exposed were confluent and they presumably have the same level of DNA damage as in cycling G_1_ phase cells. We propose that structural characteristics of cycling G_0_ phase chromatin limits the mobility of radiation-induced break ends and enhances the fidelity of DSB rejoining of non-cycling G_0_ cells in low-LET radiation.

Borgmann *et al.* reported that mitotic death is caused by lethal chromosome aberrations (CAS), such as terminal or interstitial deletions and dicentrics [30]. All these aberrations lead to a loss of DNA and, along with that, of essential genes, so that after two or three further divisions, cells irreversibly lose their proliferative capacity. Since we did not use telomere and centromere probes to analyze the chromosome aberrations, we were not sure how many cells possessed telomere deletions or dicentrics, and instead we analyzed the relationship between color-junctions, aberrations, and fragments and cell survival fraction. Correlation coefficients of 0.93 were determined for color-junction and 0.99 for aberration percent. However, the correlation coefficient was just 0.56 for fragments per cell vs survival fraction. Our results are partially supported by Wilson and Keng who reported that the final damage remaining in quiescent cells was similar to proliferating cells, and suggested that repair of DSBs is not entirely responsible for the difference in radiation sensitivity between quiescent and proliferating cells [31]. Those results imply that assumption of unrepaired DSB as a lethal event may not fit to survival fraction data when compared with different repair conditions.

Some papers have reported that high-LET damage has more ionizations and greater spatial extent, and presumably induces more complex molecular damage, with the complexity of lesions increasing with LET [32–34]. Sekine *et al.* used the same PCC technique and indicated that high-LET radiation produced complex-type DNA strand breaks [35]. Okayasu *et al.* reported that high-LET radiation induces complex DNA damage that may be difficult to repair or may not be repaired by NHEJ [36]. Our results indicated the NHEJ pathway is capable of rejoining some of the high-LET DNA damage in G_0_/G_1_ cells, but very complicated DNA damage may be beyond the capacity of the NHEJ pathway and cannot be accurately repaired even under non-cycling condition. Hirayama *et al.* reported that DNA damage induced by X-rays as well as heavy ions results from a combination of direct and indirect actions, and the contribution of indirect action to cell killing decreases with increasing LET [37]. It is possible that PLDR may be connected with DNA damage induced by the indirect action of low- to mid-LET radiations. Complex DNA damage would be induced more readily by direct action than by indirect action, thus, more complex damage is likely to be induced by high-LET heavy ions, because most of the damage results from the direct action of the ion track. It is possible that NHEJ cannot repair complex DNA damage induced by high-LET radiations accurately, resulting in absent PLDR after high-LET exposures.

The results presented in the current paper provide evidence that NHEJ functions more accurately under non-cycling than under cycling conditions after exposure to X-rays and heavy ions with low- to mid-LET values. The efficiency of PCC break rejoining detected by Giemsa staining was similar for non-cycling G_0_ and cycling G_1_ cells regardless of radiation type. However, enhanced fidelity of repair was observed under non-cycling G_0_ conditions after exposure to X-rays and 55 keV/µm silicon ions. Since DSBs rejoining in G_0_ and G_1_ phases occurs through NHEJ, similarly impaired fidelity of rejoining between the G_0_ and G_1_ condition after iron-ion irradiation suggests that DSBs induced by high-LET heavy ions cannot be repaired correctly even under non-cycling conditions, which may explain the lack of PLDR in cells exposed to high-LET iron ions.

## FUNDING

This study was supported by the research project with Heavy Ions at NIRS-HIMAC and the NASA Radiation Health Program.
